# Annual physical health checks within a forensic inpatient service

**DOI:** 10.1192/bjo.2021.232

**Published:** 2021-06-18

**Authors:** Michael Cooper, Partha Gangopadhyay

**Affiliations:** NHS Ayrshire & Arran

## Abstract

**Aims:**

Patients prescribed antipsychotics are at risk of ill effects to their physical health. Our aims were to assess whether inpatients within a forensic service, on antipsychotic medications, were receiving annual physical health monitoring in accordance with current NICE and SIGN Guidelines. Based on these Guidelines the following objectives were identified:

- 1: Physical examination, BMI and blood pressure recorded within the past year
- 2: FBC recorded within the past year
- 3: U&Es recorded within past year
- 4: LFTs recorded within the past year
- 5: HbA1C / random glucose / fasting glucose recorded within the past year
- 6: Random lipids / fasting lipids recorded within the past year

**Method:**

Inclusion Criteria: Patients admitted for longer than a year currently prescribed an antipsychotic.

Data were collected cross-sectionally on 24/7/20 for all inpatients meeting the inclusion criteria. Medical notes and the blood results system were reviewed for results of any annual physical examinations and blood monitoring over the past year.

Anonymized data were analysed using Excel.

**Result:**

13 out of 17 inpatients fulfilled the inclusion criteria. Of these 13 inpatients, 9 (69.2%) were prescribed clozapine, 1 (7.7%) zuclopenthixol, 1 (7.7%) paliperidone and 1 (7.7%) amisulpride.

All patients had BMI and blood pressures recorded within the preceding month. Only 1 patient (7.7%) had an annual physical health examination within the past year.

Findings for bloods taken within the past year were as follows:

12 patients (92.3%) had an FBC recorded

9 patients (69.2%) had U + Es recorded

9 patients (69.2%) had LFTs recorded

11 patients (84.6%) had HBA1c recorded

7 patients (53.8%) had lipids recorded

**Conclusion:**

There is scope for improvement with both annual physical examinations and blood monitoring.

All patients had regular BMIs and blood pressure recorded which is largely attributable to nursing staff protocols. Low compliance with full annual physical examination could be explained by there being no local system in place for annual physical health checks and also frequent changes in junior doctor ward cover.

Blood monitoring showed variable compliance with established standards. FBC monitoring had the best compliance, likely because the vast majority of our patients are prescribed clozapine, which necessitates minimal monthly FBC monitoring.

This audit was presented to the Forensic Team and thereafter it was agreed for a local system to be put in place for annual physical health checks in the summer each year. This will improve oportunities to optimise our patients health. We plan to re-audit at this time.

